# Preventing Healthcare-Associated Legionellosis: Results after 3 Years of Continuous Disinfection of Hot Water with Monochloramine and an Effective Water Safety Plan

**DOI:** 10.3390/ijerph15081594

**Published:** 2018-07-27

**Authors:** Maria Anna Coniglio, Margherita Ferrante, Mohamed H. Yassin

**Affiliations:** 1 Legionella Reference Laboratory, Department of Medical, Surgical Sciences and Advanced Technologies “G.F. Ingrassia”, University of Catania, via Santa Sofia 87, 95123 Catania, Italy; 2 Hygiene Complex Operative Unit, A.O.U. Policlinico—Vittorio Emanuele, via S. Sofia 87, 95123 Catania, Italy; marfer@unict.it; 3 Infection Control Department, University of Pittsburgh Medical Center (UPMC), Pittsburgh, PA 15213, USA; yassinm@upmc.edu

**Keywords:** *Legionella*, Water Safety Plan, monochloramine, risk assessment, water distribution system

## Abstract

The purpose of this study is to report the experience of the implementation and application of a 3-year Water Safety Plan (WSP) together with the secondary disinfection of water by monochloramine to control and prevent healthcare-associated legionellosis in an Italian hospital strongly colonized by *Legionella*. Risk assessment was carried out by the WSP team. The main critical control points focused on in developing the WSP for the control of *Legionella* was the water distribution system. A sampling plan for the detection of *Legionella* was implemented. A widespread contamination of the hot water distribution system by *L. pneumophila* sg5 was found. Results after 3 years of the continuous disinfection of hot water with monochloramine indicate the eradication of *Legionella*. The implementation and application of a WSP in a hospital, together with the disinfection of the water distribution system with monochloramine, can be effective in controlling the growth of *Legionella* and in preventing nosocomial legionellosis.

## 1. Introduction

*Legionella pneumophila* is a Gram-negative bacterium of public health interest for its ubiquitous presence in natural aquatic environments worldwide and for its ability to cause severe pneumonia and other infections. The bacterium replicates in water systems at an optimal temperature of 35–37 °C, but actually survives in a much wider range (4–50 °C). When inside amoeba or in biofilms, *Legionella* can resist chlorination and thus colonize artificial hot and cold-water supply systems [1]. The bacterium can also proliferate in other humid thermal habitats such as air conditioners, cooling towers, water fountains, humidifiers, whirlpool spas and hospital equipment [2].

*L. pneumophila* and its related species is the causative agent of legionellosis, which varies in severity from a mild febrile illness—the so-called Pontiac fever—to an acute and sometimes life-threatening pneumonia—the Legionnaire’s disease. It can occur as a nosocomial or travel-associated infection, sporadically or as part of an outbreak [3].

The most common route of transmission of *Legionella* is inhalation of aerosols produced from contaminated water but infection can also occur by aspiration of contaminated water [4,5,6]. It has also been documented in the exposure of babies during water births [7], while human-to-human transmission is exceptional [8]. Smokers, heavy drinkers, elderly adults and people with weakened immune systems, with chronic metabolic or lung diseases are particularly susceptible to the infection.

Hospital water distribution systems (WDSs) are more complex than residential water systems and they are more likely to be colonized with *Legionella*. Over the 2011–2015 period, 29 European countries reported 30,532 cases of Legionnaire’s disease to the European Centre for Disease Prevention and Control. Italy was among the four European countries (France, Germany, Italy and Spain) that accounted for 70.3% of all reported cases. Around 10% of all reported *Legionella* pneumonia were hospital-associated [9]. In 2015, Italy reported 82 (5.3%) hospital-associated cases, 33 (40%) of which were confirmed and 49 (60%) were probable cases, with a mortality rate accounting for 44.2% [10]. The incidence of hospital-associated or nosocomial legionellosis is increasing worldwide [11]. The patient population, potential environmental reservoirs and lack of effective prevention efforts are potential causes of nosocomial legionellosis. It is also important to consider the factors that most enhance colonization of the WDSs. These factors include water temperature and quality, biofilm formation, stagnation of the flow of water (due to construction or lack of use), use or lack of disinfectants, as well as the presence of other microorganisms that support the growth of *Legionella* [12,13,14,15].

The World Health Organization (WHO) suggests the Water Safety Plan (WSP) as the best approach to mitigate risks and hazards related to *Legionella* also in hospital settings [16]. Nonetheless, the control of *Legionella* contamination in water environments is difficult due to the capacity of this microorganism to colonize hot WDSs and resist disinfection treatments especially when inside the biofilm and amoeba [17,18,19].

Surveillance of WDSs for *Legionella* can be regarded as an important component of the primary prevention of hospital-associated legionellosis [20]. It has also been demonstrated that the WSP on healthcare settings can be useful to predict and prevent potentially critical patient’s exposure to *Legionella* [21]. Moreover, it is noteworthy that the control of biofilm is of paramount importance to avoid hospital-acquired legionellosis [11]. Among the antimicrobial agents, monochloramine (MC) is more effective for decreasing *Legionella* concentration within the biofilms in vitro as well as in model plumbing systems [22,23,24,25]. For the reasons reported above, the purpose of this study is to report the experience of the implementation and application of a 3-year WSP together with the secondary disinfection of water by MC to control and prevent healthcare-associated legionellosis in an Italian hospital strongly colonized by *Legionella*.

## 2. Methods

### 2.1. Setting

The hospital ‘Santa Marta e Santa Venera’ is a 120-bed hospital located in Acireale (Catania, Italy). Built during the period 1985–2001, the Hospital is made of one main building of 4 floors, which hosts all the wards, the staff offices and laboratories. At the lobby level there are the pharmacy as well as a restaurant. Drinking water is provided by the local municipality.

### 2.2. Samples Collection

During 2014, one year before the start of disinfection of hot water with MC, hot water samples were collected every 4 months within the WDS of the hospital. Samples were collected without flushing and without flaming in accordance with the Italian guidelines for Legionellosis Prevention [26]. One-liter sterile bottles, added with 1 mL of sodium thiosulphate in order to neutralize residual free chlorine were used for bacteriological analyses. According to ISO 19458, procedures for sample collection, transport and storage were established. Bottles were returned to the laboratory immediately after sampling for bacteriological analyses [27].

### 2.3. Microbiological Analyses

Isolation of *Legionella* was performed in accordance with standards procedures ISO 11731:1998 [28]. One liter of water from each sampling point (taps and showers) was concentrated using 0.2 µm polycarbonate filters (Sartorius Stedim Biotech GmbH, Goettingen, Germany). Each membrane was vortexed for 15 min in 10 mL of the correspondent water sample to detach bacteria. For each water sample, an aliquot of 5 mL of the concentrated sample underwent immediate cultural examination while the remaining 5 mL was treated with heat by exposure to 50 °C for 30 min. From both the samples (concentrated and heat-treated sample), aliquots of 0.1 mL were transferred onto one plate of Buffered Charcoal Yeast Extract (BCYE) agar (Charcoal Yeast Extract, CYE, agar with added BCYE*α* growth supplement, Oxoid Ltd., Basingstoke, Hampshire, UK) and 0.1 mL onto one plate of Glycine Vancomycin Polymyxin Cycloheximide (GVPC) agar (BCYE agar with added GVPC selective supplement, Oxoid). Plates were incubated at 37 °C within jars containing modified atmosphere (2.5% CO_2_). After 4, 8 and 14 days of incubation, colonies suggestive for *Legionella* grown on BCYE and GVPC were confirmed on the basis of cultural testing (lack of growth on CYE agar) and were subjected to identification using a multi-purpose latex agglutination test with polyvalent antisera (Legionella Latex Test, Oxoid) and monovalent antisera (Biogenetics Srl, Tokyo, Japan).

Results were expressed in cfu/L and the counts referred to water samples concentrated 100 times (1 L in 10 mL of the water sample). The detection limit of the culture procedure was 100 cfu/L.

### 2.4. Water Piping and Treatment

A thorough analysis of the water systems was carried out with the facility manager, the engineering company and the *Legionella* experts. The main findings of this analysis are discussed below.

Chlorinated drinking water (chlorine residual level 0.1–0.2 mg/L) is pumped from municipality pipework, and once it enters the hospital, it is filtrated and softened (total hardness after softening 15°F). Pipe distribution from the municipality to the storage tanks is made of galvanized steel while the storage tanks are made of stainless steel. After the pumping station the water piping is made of stainless steel. Water piping is mainly made of crimped AISI 316L stainless steel for risers and return loop and PP-r (random polypropylene) plastic for the end distribution from the risers to the outlets of each room.

The WDS of the building is made of risers that brings water to each floor. A horizontal loop distributes water to all the outlets. The building is equipped with one hot water production unit. Hot water is produced at 70 °C on a plate heat exchanger and is accumulated in eight 500 L thermal storage tanks in parallel. Although the contemporary use of the hot water from all the outlets is unlike, assuming that the average water usage is of 100 L/day per bed, the estimated retention time of the storage tanks is of 8 h. A mixing valve is used to lower the temperature of the water at the taps and showers between 48–50 °C, as decided by the medical and nursing staff to avoid scalding. Anyway, in Italy, 48 ± 5 °C is mandatory temperature of the distributed hot water for public buildings (DPR 412/93). 

The hot water heater delivers water to the main loop at level 1, from which the risers depart up to level 4. The recirculation loop at level 3 collects all the flows in the risers and directs them toward the hot water production units. 

## 3. Results

In total, 481 water samples were collected during 2014 and a systemic colonization of the hot water networks was demonstrated. In particular, *L. pneumophila* sg5 was isolated from 100% of the sampling points (mean count ranging from 10^3^ to 10^5^ cfu/L). On the contrary, *Legionella* was not isolated in the 8 cold water samples collected. In particular, a sampling point was installed just downstream of the softener to enable water samples to be collected and to be tested for the aerobic colony count and legionellae. The aerobic colony count was compared with that of the incoming mains supply water. There was no evidence of colonization and *Legionella* was not detected. Thus, it was assessed that there was no need to disinfect the softener as this may shorten the life of the resin bed. Anyway, it was decided to sample the softener at least once a year. 

When *Legionella* is recovered at concentrations >10^3^ cfu/L in more than 20% of the samples, Italian guidelines for Legionellosis Prevention establish that disinfection must be put in place and clinical surveillance must be implemented at least for patients at higher risk [26]. Thus, it was clear that a continuous disinfection method had to be adopted together with a systematic clinical surveillance performing laboratory diagnostic tests on suspected cases.

### 3.1. Water Safety Plan (WSP) Team

The WSP team was assembled at the beginning of 2015. It was made up of the hospital risk managers, the medical director, the infection control team, the facility manager, the engineering company, the contractors and proven experts in *Legionella* remediation. The Legionella Reference Laboratory did not take part to the WSP implementation but was involved in the validation step and audited the hospital several times.

### 3.2. WSP Implementation

**Figure 1:**
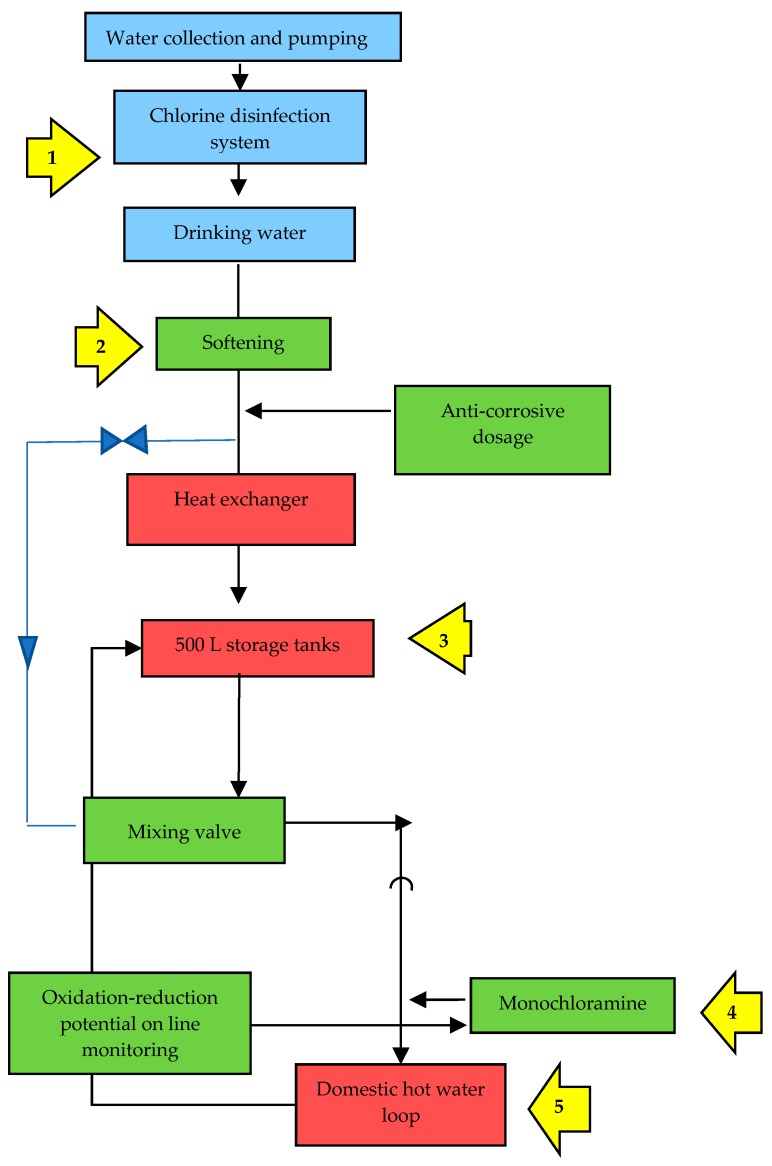
The main critical control points (CCPs) for the control of *Legionella* identified at the hospital ‘Santa Marta e Santa Venera’ throughout the water distribution system.

According to the indications of the WHO [29], the implemented WSP consisted of 4 main steps: system assessment, monitoring, management and communication, surveillance. As shown on Table 1, the ‘system assessment’ step provided a detailed and systematic assessment and prioritization of hazards, as well as operational monitoring of barriers and control measures. The main critical control point (CCP) was the WDS (Figure 1). The ‘monitoring’ step focused on the identification and monitoring of control measures used to ensure water safety (e.g., disinfectant levels, flushing protocol, temperature, etc.). The step ‘management and communication’ documented the system assessment and monitoring and described actions to be taken during normal operation and in case of incidents (e.g., notification of a case of nosocomial Legionnaire’s disease, remedial actions after adverse monitoring results, etc.), including documentation and communication (e.g., communication procedures that should be followed by any person involved in the WSP implementation). Finally, the step ‘surveillance’ provided an indication of the overall performance of the WSP through the systematic collection and analysis of data to verify if control measures applied are operating properly (e.g., audits to confirm that operational monitoring and corrective actions are being undertaken as stated in the WSP, periodically sampling for legionellae in water at the taps, etc.). 

Finally, in the choice of the water disinfection method against *Legionella* particular attention was focused on piping materials and water quality. Taking into consideration that the WDS of the hospital is made predominantly of crimped AISI 316L stainless steel and PPr, the use of chlorine dioxide for the secondary disinfection of the water was not considered because the compatibility with piping material was not assured, namely for hot water [30,31]. Thus, it was decided to use MC for hot WDS due to the high material compatibility of this molecule [32]. Moreover, piping corrosion caused by a disinfectant can be affected by many characteristics of the water itself and the combinations thereof including pH, alkalinity, temperature, natural organic matter, and the type of scale that is formed [33]. The chemical and physical analysis of water (data not shown) showed that it respected the limits and ranges from Italian Legislation [34] and that it could be compatible with MC. Thus, the hot water was softened and treated with MC with a patented system (Sanikill, Sanipur S.R.L., Italy) which is specially designed to treat domestic hot water without accumulation of by-products (NH_4_^+^, THMs, NDMA) [35] and which avoids side reactions (e.g., dichloramine formation). Desired residual MC level was 2–3 mg/L.

### 3.3. Preliminary Legionella Remediation

Taking into consideration the widespread contamination of all the WDS by *L. pneumophila* sg5, two different protocols for the preliminary remediation of the hot WDS were carried out. The protocols for remediation were as follows. 

Each protocol was based on the use of the existing disinfection equipment and did not contemplate the addition of further products.

First, a chlorine hyper-dosage was applied so that residual chlorine at distal outlets was about 1 mg/L. Flushing was carried out for 5 min for each outlet, subsequently the system was kept with this concentration for 1 h before reducing chlorine residual concentration at 0.1–0.2 mg/L with another thorough flushing. 

Second, a hyper-dosage of MC produced in-situ was applied at the hot water production station. MC concentration was raised at 5 mg/L before thorough flushing of each outlet for 5 min. Moreover, hot water storage tanks bottom valves and return loop valves were fluxed for 5 min. MC concentration was kept at this level for 1 h and then it was restored at 2 mg/L residual level with another thorough flushing. Thanks to the continuous disinfection with MC, the temperature of the produced hot water was lowed at 60 °C, with a saving on energy costs of 2219.00 €/year.

After each remediation, water samples were randomly collected to confirm the eradication of *Legionella* in all the WDS. Sampling was carried out as described above. After the shock treatment all the samples were negative for *Legionella*.

After the start of continuous disinfection with MC, from April 2015 to April 2018, all the 481 outlets previously sampled were sampled again. Water samplings were carried out every 4 months and all the samples were negative for *Legionella*. Only positive samples >10^3^ were considered because the Italian guidelines recommend disinfection only for a *Legionella* count >1000 cfu/L in hospitals without documented cases of disease (Table 2).

### 3.4. Chemical Analyses

For each water sample MC and free ammonia were determined by a modified indophenol method (Monochlor-F, Hach, Hach DR/900, Loveland, CO, USA). Free and total chlorine were determined by the *N*,*N*-diethyl-*p*-phenylendiamine (DPD), while nitrate and nitrite were determined by specific colorimetric methods (Hach DR/900).

Oxidation-reduction potential (ORP) of hot water was monitored on-line with a hot water ORP probe (Tecme S.r.l., Trento, Italy) mounted on the Sanikill device.

As shown on Table 3 the levels of ammonium, nitrites and nitrates did not exceed their limits during the period April 2015–October 2017.

## 4. Discussion

The present article outlines the process involved in developing a WSP to control and prevent nosocomial legionellosis in an Italian hospital whose domestic hot water, heavily contaminated with *L. pneumophila* sg5, was additionally disinfected with MC. Control measures to minimize *Legionella* proliferation ranging from source of water quality, treatment of source water and piping materials were specifically considered.

Special precautions are required to prevent and control the presence of *Legionella* in hospitals, because aerosols from showers, medical devices and cooling systems are a route of infection and those facilities contain high-risk populations. In hospitals contaminated with *L. pneumophila* non-sg1 the risk of developing hospital-associated legionellosis is very low. However, isolated cases and outbreaks have been related to *L. pneumophila* sg5. In 2005, a nosocomial outbreak caused by *L. pneumophila* sg5 was reported in Finland [36]. In 2007, a case of nosocomial legionellosis due to *L. pneumophila* sg5 was reported in an Italian hospital whose WDS was contaminated by both *L. pneumophila* sg1 and sg5 and where nosocomial *Legionella* infections had never been previously detected [37]. In 2008, a fatal nosocomial *L. pneumophila* sg5 infection was reported in a French patient with leukemia [38]. More recently, in 2016, a nosocomial outbreak of *L. pneumophila* sg5 was associated with two heat exchangers in Canada [39]. Taking into consideration literature data reporting nosocomial cases related to *L. pneumophila* sg5 and considering the heavy contamination of the hospital’s WDS by this serogroup, it was decided to adopt a preventive approach based on the application of a WSP and the continuous disinfection of the hot WDS, although nosocomial Legionnaire’s disease cases had never been previously detected in that hospital.

Different strategies for preventing hospital-associated legionellosis can be adopted. All of them take into consideration three main elements: (*i*) environmental surveillance, (*ii*) clinical surveillance, and (*iii*) intervention threshold levels. The Allegheny County Health Department Pennsylvania, US, advocates environmental monitoring for *Legionella* in hospital’s WDS together with pneumonia surveillance [40]. The CDC strategy proposes intensive clinical surveillance without routine environmental surveillance, except in Transplant Units [41]. Italian guidelines for prevention and control of legionellosis set intervention threshold levels for WDS based on the *Legionella* load detected in water samples [42]. It is noteworthy that the presence of *Legionella* in a hospital’s WDS does not necessarily lead to legionellosis [43,44]. Conversely, a negative result is no guarantee that *Legionella* is not present in the system. The constant environmental and clinical monitoring, therefore, together with the respect of threshold levels, are important elements of the control of hospital-acquired Legionnaire’s disease but they are not the only ones. It seems to be more useful to combine environmental monitoring with a risk assessment based on all potential hazards that may arise in maintaining the hospital’s WDS.

As suggested by the WHO, the preferred approach to health risk assessment in evaluating specific risks of exposure to *Legionella* from a WDS is to develop a WSP, which provides a systematic assessment and prioritization of hazards, as well as operational monitoring of barriers and control measures [45]. In particular, the WSP incorporates a series of ‘multiple barriers’ specifically designed to minimize the transmission of *Legionella* from the source of water to the users’ tap. Risk minimization mainly depends on good temperature control and on water treatment with chemicals because *Legionella* is especially resistant to the most common used disinfectants. Thus, the appropriate selection, design and operation of technologies are essential to ensure a successful treatment of the water.

At the moment, chemical disinfection is the most reliable approach for disinfection of hospital drinking water. In our study, it was decided to use MC for hot WDS due to the high material compatibility of this disinfectant and because literature data suggest that hospitals supplied with drinking and hot water treated with MC are less likely to have a reported outbreak of Legionnaires’ disease [18]. Results of water samplings carried out during a 3-years period show that all the samples were negative for *Legionella*. The WHO Guidelines for Drinking-water Quality indicates that the water temperature should be maintained outside the range of 25–50 °C, at which *Legionella* proliferates [16]. The use of MC gave the possibility to low the hot water temperature from 70 °C to 60 °C, with a conspicuous saving in energy costs, thus demonstrating that the technology adopted is also a cost-effective solution. 

Our study is subject to at least two main limitations. First of all, the observed decrease in WDS contamination cannot be attributed only to MC but rather to the sum of actions that were taken to eradicate the contamination (chlorine hyper-dosage, hyper-dosage of MC, flushing). Thus, the effectiveness of the disinfectant needs to be carefully evaluated in the long run, mainly because it has been recently reported the emergence of viable but non-culturable (VNBC) *Legionella* during a long period of continuous MC treatment of a hospital water network [46]. Secondly, estimated energy savings in the present study was of 2219.00 € per year. Nonetheless, the costs related to the continuous disinfection with MC and to the water samplings should have been assessed and weighed against the costs associated with legionellosis hospitalization (€ 27.54/episode) and the elevated mortality rate.

## 5. Conclusions

As far as we know, our study is the first report about the application of a WSP together with the disinfection of the WDS with MC in controlling *Legionella* growth.

The implementation and application of the WSP together with the disinfection of the WDS with MC can be effective in controlling *Legionella* growth and in preventing nosocomial legionellosis.

## Figures and Tables

**Table 1:** The four steps of the Water Safety Plan (WSP) for the control of *Legionella* implemented at the Hospital ‘Santa Marta e Santa Venera’.

**System assessment**	*Description of the system*	
	Description of the system focused on water piping, hot water production, at risk devices, water uses.	
	*Identification of risks* Risk areas were not ranked into different levels. Risk level was considered high in all the wards as all patients were considered potentially at high risk.	
	*Identification of the operational monitoring barriers*	
	Disinfection of the WDS with MC. Ensuring that the system operates safely and correctly and is well maintained. Yearly visual inspection of the accessible parts (storage tanks, distal outlets) of the WDS for damage and signs of possible contamination. If debris or scales or biofilm are found, then the inspection must be carried out more frequently. Scale control by maintaining the cleanliness of the water softener. Routine cleaning procedures for distribution system (storage tanks, distal outlets). Semesterly draining of the hot water storage tanks. Weekly flushing for several minutes of taps and showers when they are not in regular use. Use of sterilized water to clean respiratory equipment. Cleaning and disinfection protocols for respiratory equipment.	
	*Identification of the control measures* Checking the performance of the disinfection system (MC at 2 mg/L residual level): daily monitoring of oxidation-reduction potential on-line monitoring of hot water with an ORP probe mounted on the device; monthly determination of MC, free and total chlorine; semesterly determination of free ammonia, nitrite and nitrate. Temperature control for hot and cold-water systems. Monthly heterotrophic colony counts at the storage tanks and distal outlets as an indication of whether microbiological control is being achieved. Four-monthly sampling and testing for the presence of *Legionella* in the WDS and at the point of use to show that adequate control is being achieved.	
	*Assessment of the water distribution system* CPPs and permanent monitoring.	
**Monitoring**	Identification of control measures	Flushing of the outlets, preliminary piping sanitization, continuous disinfection with MC.
	Monitoring control measures	*Legionella* surveillance, determination of ammonium, nitrites and nitrates levels, check of temperature, water quality (including ORP on line monitoring), residual disinfectant concentration, flushing protocol, maintenance procedures.
**Management and communication**	Development of supporting programs	Medical and nursing staff, technical and housekeeping staff were trained to actively participate to the WSP implementation tasks.
	Preparation of management procedures	Different levels of risk were considered, depending on various water system parameters. *Situation under control* (*Legionella* < 1.000 ufc/L; cold and hot water temperature at the outlets <22 °C and 48–50 °C, respectively; MC at 2 mg/L residual level; free ammonia < 0.50 ppm; nitrites < 0.50 ppm; nitrates < 50 ppm). *Alertness* (*Legionella* between 1.000–10.000 ufc/L; cold and hot water temperature at the outlets >22 °C and <48–50 °C, respectively; MC at <2 mg/L residual level; free ammonia > 0.50 ppm; nitrites > 0.50 ppm; nitrates > 50 ppm). *Alert threshold* (*Legionella* > 10.000 ufc/L). Management procedures were prepared by the risk manager and the facility manager with the support of the *Legionella* experts. *Situation under control*: no action required. *Alertness*: correction of water temperature; correction of MC dosing. *Alert* threshold: hyperchlorination (20–50 mg/L for 1–2 h, respectively); raising of the MC concentration (5 mg/L for 1 h); flushing of each outlet for 5 min.
	Documentation and communication procedures	Medical directors and the risk manager supported by the *Legionella* experts established procedures that should be followed by any person involved in the WSP implementation. Documentation management followed ISO9001:2008 principles.
**Surveillance**	Validation of the WSP	*Legionella* sampling and analysis were accepted as validation for the effectiveness of the implemented WSP.
	Sampling program	Sampling program included a sampling campaign after preliminary sanitization and continuous monochloramination.
	Auditing	Several audits were carried to validate the WSP measures. After the start of continuous disinfection with monochloramine new audits were carried out to assess the actual implementation of the WSP measures and define the improvement plan.
	System assessment	Reviewed every year and after any major changes to the WDS or management (e.g., changes of water quality; engineering changes).

**Table 2:** *Legionella pneumophila* sg5 contamination of the drinking and hot water distribution systems (WDS) before and after the start of continuous disinfection with monochloramine (2–3 mg/L).

Water Distribution System	Positive > 10^3^ cfu/L N (%)	
	Before	After
Cold water	0/8 (0%)	0/8 (0%)
Hot water		
Taps	221/221 (100%)	0/221 (0%)
Showers	220/220 (100%)	0/220 (0%)
Storage tanks	32/32 (100%)	0/32 (0%)

**Table 3:** Levels of ammonium, nitrates and nitrites at base line and during the treatment of the WSP with monochloramine.

Month/year	Nitrates NO^3−^ (Limit 50 ppm)	Nitrites NO^2−^ (Limit 0.50 ppm)	Ammonium NH^4+^ (Limit 0.50 ppm)
April 2015 (before MC disinfection)	17.0	0.025	0.05
October 2015			0.48
April 2016	9.6	0.020	0.32
October 2016			0.29
April 2017	4.6	0.040	0.25
October 2017			0.21
